# Host Transcriptomic Response Following Administration of Rotavirus Vaccine in Infants’ Mimics Wild Type Infection

**DOI:** 10.3389/fimmu.2020.580219

**Published:** 2021-01-21

**Authors:** Alberto Gómez-Carballa, Ruth Barral-Arca, Miriam Cebey-López, Maria José Currás-Tuala, Sara Pischedda, José Gómez-Rial, Dominic Habgood-Coote, Jethro A. Herberg, Myrsini Kaforou, Federico Martinón-Torres, Antonio Salas

**Affiliations:** ^1^ Genetics, Vaccines and Pediatric Infectious Diseases Research Group (GENVIP), Instituto de Investigación Sanitaria de Santiago (IDIS) and Universidad de Santiago de Compostela (USC), Santiago de Compostela, Spain; ^2^ Translational Pediatrics and Infectious Diseases, Department of Pediatrics, Hospital Clínico Universitario de Santiago de Compostela, Santiago de Compostela, Spain; ^3^ Unidade de Xenética, Instituto de Ciencias Forenses (INCIFOR), Facultade de Medicina, Universidade de Santiago de Compostela, and GenPoB Research Group, Instituto de Investigacinó Sanitaria (IDIS), Hospital Clínico Universitario de Santiago (SERGAS), Santiago de Compostela, Spain; ^4^ Section of Pediatric Infectious Diseases, Imperial College London, London, United Kingdom

**Keywords:** biomarkers, RNA-seq, transcriptomics, vaccination, miRNA, rotavirus, machine learning, intussusception

## Abstract

**Background:**

Rotavirus (RV) is an enteric pathogen that has devastating impact on childhood morbidity and mortality worldwide. The immunologic mechanism underlying the protection achieved after RV vaccination is not yet fully understood.

**Methods:**

We compared the transcriptome of children affected by community-acquired RV infection and children immunized with a live attenuated RV vaccine (RotaTeq^®^).

**Results:**

RV vaccination mimics the wild type infection causing similar changes in children’s transcriptome, including transcripts associated with cell cycle, diarrhea, nausea, vomiting, intussusception, and abnormal morphology of midgut. A machine learning approach allowed to detect a combination of nine-transcripts that differentiates vaccinated from convalescent-naturally infected children (AUC: 90%; 95%CI: 70–100) and distinguishes between acute-infected and healthy control children (in both cases, AUC: 100%; 95%CI: 100–100). We identified a miRNA hsa-mir-149 that seems to play a role in the host defense against viral pathogens and may have an antiviral role.

**Discussion:**

Our findings might shed further light in the understanding of RV infection, its functional link to intussusception causes, as well as guide development of antiviral treatments and safer and more effective vaccines. The nine-transcript signature may constitute a marker of vaccine protection and helps to differentiate vaccinated from naturally infected or susceptible children.

## Background

Infectious acute gastroenteritis is one of the major causes of hospitalization in children, with rotavirus (RV) being the most frequent etiologic agent in severe disease (1). RV is also one of the leading causes of infant death in developing countries; it was estimated that RV was responsible for the death of more than 600,000 children per year worldwide before the introduction of vaccines, and 128,000 after the introduction of vaccines in children younger than five years (2–4). As there are no antiviral therapies available, the treatment of RV infection is based on avoiding dehydration and replacing the electrolyte losses of affected children. The development and introduction of RV vaccines have resulted in significant fewer cases of severe gastroenteritis in those countries where RV vaccination is included in the routine schedule (5, 6).

Two different vaccines are licensed in Europe for the immunization against RV: (a) the live attenuated pentavalent human-bovine reassorted vaccine RotaTeq^®^ (RV5, Merck and Co, Inc, Pennsylvania, USA), and (b) the live attenuated human vaccine Rotarix™ (RV1, GSK Biologicals, Rixensart, Belgium) (7). RV5 is composed of a combination of five human/bovine reassorted RV that replicate poorly in the gut (3). RV1 is made from a single human live attenuated strain that replicates easily in the intestine (3, 7). Both vaccines confer protection and have shown real-life effectiveness and impact; however, the exact immunologic mechanism conferring protection against RV gastroenteritis is not fully understood (8). The development of future RV vaccines or the improvement of current formulations is limited by our incomplete knowledge of the mechanisms responsible for RV pathogenesis and the host susceptibility (9). Possible heterologous effects of RV vaccination are also the focus of attention (see (10–12) and references therein). It has been recently reported that RV infection is able to provoke global changes in the transcriptome of infected cells to evade the innate host response; likewise, the host develops mechanisms to avoid viral invasion, including a strong inhibition of glycophorin genes (13).

Despite the importance of these interactions and the burden that RV means to human health, only a few human blood gene expression studies have been published to date (13, 14); none of them have investigated how vaccines influence the blood transcriptome. There is therefore a lack of knowledge on how RV interacts with the host (13) and the mechanism that underlies the acquired immunity after RV vaccination.

To the best of our knowledge, this is the first transcriptomic investigation of RV vaccine response in whole blood, and we present a comparison of vaccinated infants *vs.* wild type RV infected children and age-matched healthy controls.

## Methods

### Samples and Ethical Approval

**Figure 1:**
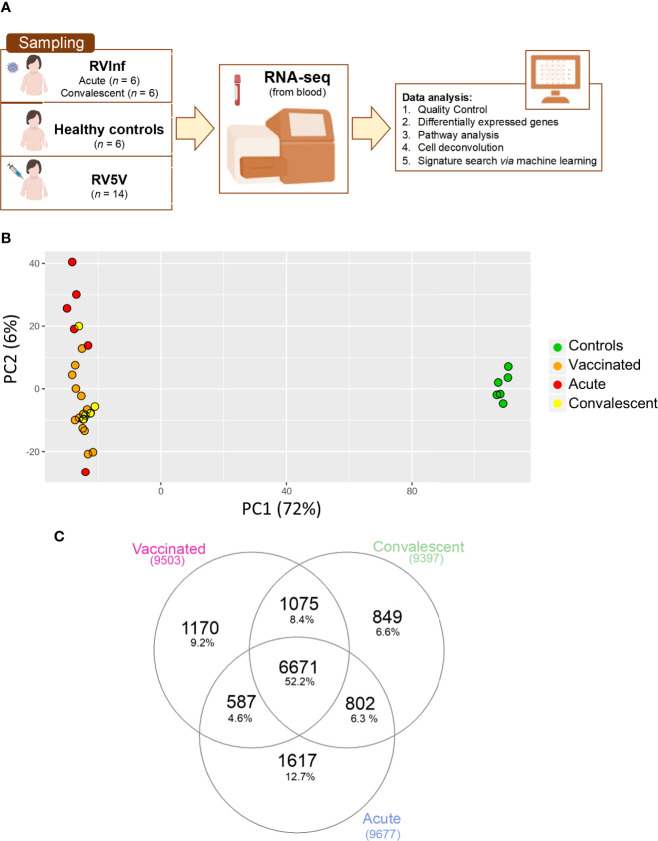
**(A)** Scheme of sampling and project design; **(B)** Principal component analysis (PCA) built with the top 500 most highly expressed genes; **(C)** Venn plot of the DEGs when comparing healthy controls *vs*. vaccinated children and healthy controls *vs.* acute and convalescent infected (community-acquired) children, corrected by age and gender.

The Spanish cohort of 32 western-European children, prospectively collected between 2013 and 2014 at the Hospital Clínico Universitario of Santiago de Compostela (Galicia; Western Spain) (**Figure 1A**) comprised: (*i*) six healthy age-matched controls (with all the vaccines of the Spanish immunization schedule up to date but no rotavirus vaccine), (*ii*) 14 RV5 vaccinated infants, *i.e.*, all the regular vaccines up to date plus three RV5 doses (RV5V group), and (*iii*) six RV infected children required medical attention due to moderate or severe symptomatology (RVinf group) at two different time-points, namely, acute (during medical attendance) and convalescent phases (40 ± 10 days after clinical recovery) (**Table S1**). A blood sample was obtained from these children using a PAXgene RNA tube (PreAnalytiX GmbH). Ages ranged from nearly 2 to 34 months (male/female ratio = 0.77). The mean time elapsed from hospital admission to blood collection in infected children was three days; whereas, in RV vaccinated children the blood sample was taken prior to vaccination and one month after the last RV5 dose. There were no remarkable clinical features in the individuals recruited. A subset of these controls and infected children were previously analyzed in (13). We used RV5 in our study instead of RV1 because it was the only RV vaccine available in Spain at the time of sample collection (2013-2014) (15).

All researchers were specifically trained in the study protocol for patient recruitment, sampling processing, and storage. The study was conducted following the principles of Good Clinical Practice and of the Declaration of Helsinki. Written informed consent was obtained from a parent or legal guardian for each subject before study inclusion. The project was approved by the Ethical Committee of Clinical Investigation of Galicia (CEIC ref. 2012/301).

### Quality Control of Total RNA, Library Preparation, and RNA-Seq

We followed the same quality standards described in Salas et al. (13). Bioanalyzer 2100 and Qubit 2.0 were employed to evaluate the quality and the quantity of the collected RNA. Globin mRNA (which can make up to about 70% of the mRNA in blood) can compromise the detection of other specific mRNAs from leukocytes. We reduced the amount of globin RNA using GLOBINclearTM-Human Blood Globin Reduction Kit (Life Technologies; CA, USA) to obtain a clearer signal from mRNAs from leukocytes. Then, Poly(A) + RNA was isolated on poly-T oligo-attached magnetic beads and chemically fragmented prior to reverse transcription and cDNA generation. The cDNA fragments subsequently went through an end repair process, the addition of a single ‘A’ base to the 3′ end, and then ligation of the adapters. Finally, the products were purified and enriched with PCR to create the indexed final double stranded cDNA library. High sensitivity assay and quantification of libraries were determined by real-time PCR in LightCycler 480 (Roche). Equimolar pooling of the libraries was performed before clusters’ generation. Clonal clusters from single molecule DNA templates were created using *cBot* (Illumina). The *cBot* system isothermally amplifies cDNA fragments covalently bound to the flow cells to create hundreds of millions of clusters, with around ~1,000 identical copies of a single template. An Illumina HiSeq 2000 sequencer was used to sequence the pool of cDNA libraries using paired-end sequencing (100 × 2).

### RNA-Seq Bioinformatic Analysis

RNA-seq quality data analysis was carried out following the recommendations described in Conesa et al. (16). We first performed the quality control of the raw data from single samples using *FastQC* (17) to ensure the optimal quality of the reads and avoid potential technical biases due to low quality samples in the dataset which may affect the downstream analysis. Next, FastQC output from single samples were analyzed together using *MultiQC* (18) to create a single report across the samples. Afterward, the whole transcriptome paired-end reads were mapped against the human genome provided by Ensembl v. GRCh37_r87/release 87 using the aligner *STAR* (https://github.com/alexdobin/STAR). We used *STAR* to generate the raw count expression matrix with the number of reads that map to each gene. Normalization of raw data is an essential step to obtain comparable samples, and it is of key importance to accurately interpret the results in transcriptomics. For this reason, we tested different normalization methods with the raw count data using R v3.4.3 (http://www.r-project.org), including the following: Reads Per Million Mapped reads (RPKM) (19) and Trimmed Means of M values (TMM) (20) both implemented in the *edgeR* package (21); and Conditional Quantile Normalization (CQN) (22) and *Deseq2* (23) using the library *tweeDEseq* package (24).

As all tested normalization methods yielded virtually the same result, we choose the one implemented in *Deseq2* since it is a well-known and popular tool for differentially expression gene analysis of RNA-seq data. *Deseq2* package was also used to perform the differential expression analysis.

The samples were previously analyzed for their ancestral background in Barral-Arca et al. (25) indicating their main European ancestry, then matching the self-reported ethnicity.

### Statistical Analysis

In order to obtain differentially expressed genes (DEGs) between cohorts we used the Negative Binomial distribution (20) implemented in the *DESeq* package. Gender and age were included in the model as known covariates in order to account for differences in gene expression from age and sex related genes. In addition, we also used the Surrogate Variable Analysis (SVA) method implemented in the *sva* R package to estimate potential hidden and unwanted variation that might be affecting many or all of the genes in the dataset. The surrogate variables obtained from the analysis were also used as covariates in the model to adjust for unknown or unmodeled sources of noise. A generalized linear model was fitted in each cohort, and a t-statistic was calculated for each gene. *P*-values were corrected for multiple testing using the Benjamini-Hochberg false discovery rate (FDR) approach.

We used Principal Component Analysis (PCA) to visualize the global transcriptome patterns of RNA-seq data and to identify outliers. PCA was undertaken using the *DESeq2* R package. In addition, we carried out a Permanova analysis as implemented in the *vegan* R package to assess statistical differences between clusters.

We also carried out an over-representation and pathways analysis using the DEGs obtained from different comparisons through a hypergeometric test that calculates the probability that the proportion of genes within a given function/pathway might be found by chance within our selection of genes. We used two different public databases: (*i*) the Gene Ontology Project [GO (26)], and (*ii*) the Kyoto Encyclopedia of Genes and Genomes or KEGG (27). Ingenuity Pathway Analysis (IPA; https://www.qiagenbioinformatics.com/) tool was used to estimate the most significantly altered pathways and generate networks of biomarkers. Among the DEGs between RV5V and controls, we focused on those reported to be associated with intussusception according to the Disgenet database (28), namely: *STK11*, *PTEN* and *ARID1B*. We also investigated the *APC* gene as it was also reported to be associated to intussusception in the literature (29).

The R package *CORNA* (30) was used to investigate if microRNAs (miRNAs) can be regulating mRNA expression levels between the genes differentially expressed in vaccinated children.

The two-way hierarchical clustering analysis heatmaps of the genes associated with nausea, vomiting, and diarrhea according to Ingenuity^®^ and the genes associated with hsa-mir-149 according to CORNA were generated using hierarchical clustering and the R package *gplots*.

We used a linear discriminant analysis to identify a transcript signature that distinguishes unvaccinated children from vaccinated children using Parallel Regularized Regression Model Search or PReMS (31). The ability of the predicted model to discriminate vaccinated children was assessed by computing the Area Under the Curve (AUC) and the sensitivity, and the specificity at the optimal cutpoint according to the Youden index was calculated with the R package *Optimal Cutpoints* (32). PReMS was initially built splitting the whole dataset into a training set (80% of the samples) and a test set (20% of the samples taken at random).

The performance of the proposed signatures was evaluated using Receiver Operating Characteristic (ROC) curves that represent the true positive rate (TPR) against the false positive rate (FPR) at different threshold cutpoints. ROC curves were built in R using the package *pROC* (33).

Boxplots were built using the R package *beeswarm* (https://cran.r-project.org/web/packages/beeswarm) to represent the total score for the transcript signature in the different groups analyzed. The total score was obtained using the same approach as the described in (34–36) for Disease Risk Score (DRS) calculation.

The proportions of different cell types in peripheral blood may differ naturally, and in consequence, mRNA measurements can vary as well (37). We used the Cell-type COmputational Differential Estimation (CellCODE) (38) method implemented in the R package of the same name, which assigns expression alterations to their cell type of origin with high accuracy, to analyze if there were any difference between the cell-type proportions in the blood of our three groups under study.

The data generated in this study have been deposited in the European Nucleotide Archive (ENA) at EMBL-EBI under accession number PRJEB41347 (https://www.ebi.ac.uk/ena/browser/view/PRJEB41347).

## Results

### RNA-Seq Results

To study the changes experienced in the transcriptome of RV5V and RVinf we performed large-scale expression screening using RNA-seq. A PCA of the whole transcriptome identified one outlier among the acutely infected children, which was eliminated from the followed-up analysis. After eliminating this outlier, the first principal component of the PCA (PC1; accounting for most of the variation, 72%; **Figure 1B**), shows two main significant clusters (Permanova *P*-value = 0.001) separating healthy controls from vaccinated children plus infected children, suggesting that both RV wild type and the vaccine attenuated virus modify the global transcriptome in a similar manner.

We obtained 9,503 DEGs in the vaccinated *vs.* controls comparison, and 8,958 in the infected children (RVinf acute phase and RVinf convalescent phase) *vs.* controls (**Table S2**, **Figure 1C**). It is interesting to note that more than half (~52%; **Figure 1C**) of the DEGs of vaccinated children against healthy controls overlap with those differentially expressed in infected children against healthy controls (**Figure 1C**).

**Figure 2:**
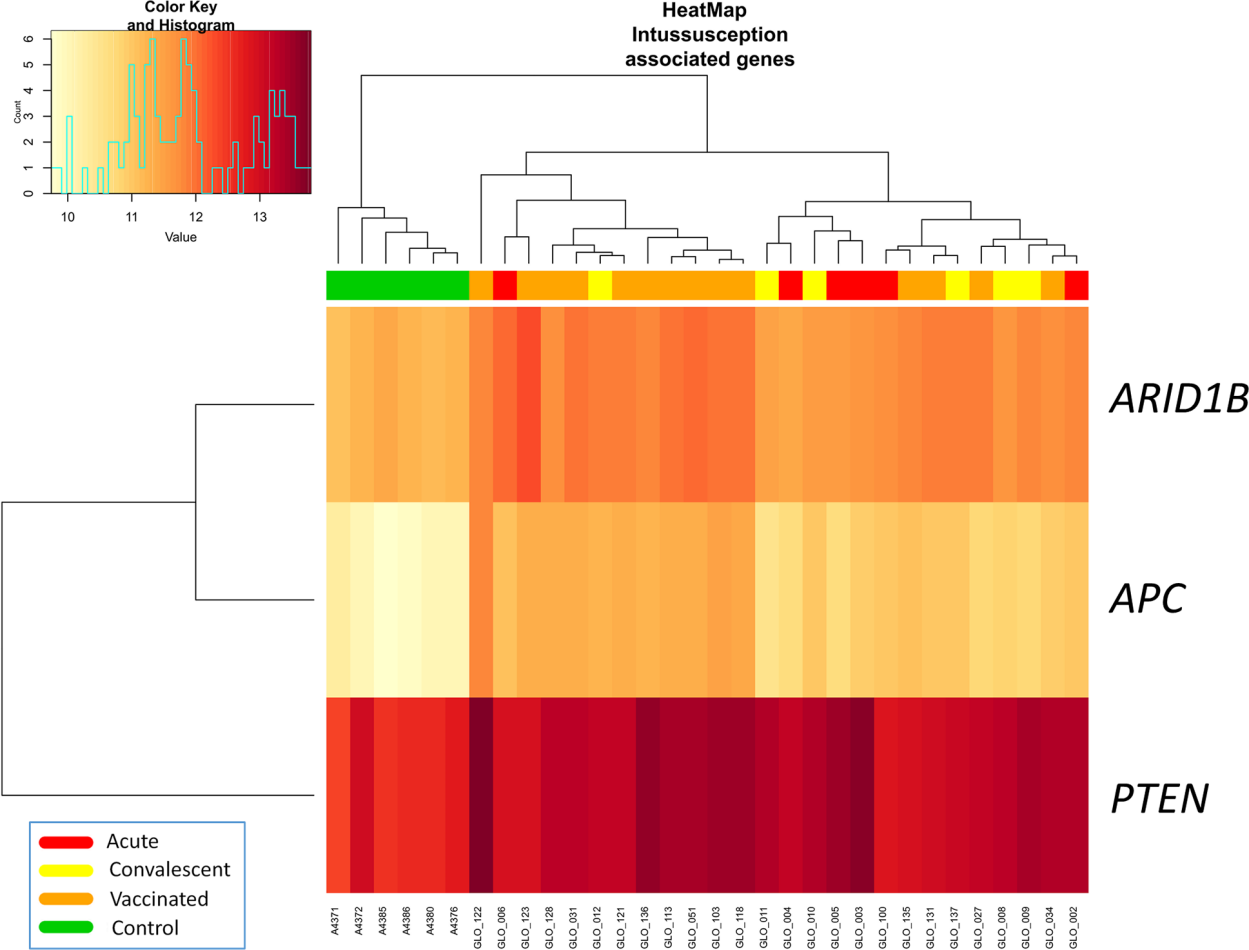
Two-way hierarchical clustering analysis heat map of DEGs associated with intussusception according to IPA. Each row represents one transcript; each column represents one patient, with a red bar above indicating the sample status red (acute), yellow (convalescent), orange (vaccinated), green (control). Expression intensity is indicated by color (high expression in red; low expression in yellow).

Three out of four genes related to intussusception (*ARID1B*, *APC*, *PTEN, and STK11*) according to Disgenet database were significantly differentially expressed between RV5V and controls (**Figure 2**). The three genes were up-regulated in the RV5V group: *ARID1B* lLog Fold Change [logFC]: 0.76; *P*-value 2.1 × 10^−11^), *PTEN* (logFC: 0.64; *P*-value = 3.7 × 10^−5^), and *APC* (logFC: 1.32; *P*-value = 7.7 × 10^−14^).

RVinf convalescence samples have a large number of DEGs when compared against controls, showing a persistence of transcriptomic signals even after clinical recovery. It has been recently reported that viruses can stimulate the immune system and affect gene expression during long periods (39).

We detected 480 DEGs when comparing RVinf acute phase and vaccinated group, whereas only 25 were found when comparing RVinf convalescent phase with vaccinated, pointing to a similar systemic transcriptomic pattern generated by the vaccine and the virus in the convalescence phase of the disease. Only 80 out of the 480 DEGs between RVinf acute phase and vaccinated children (**Table S2**) were over-expressed (12 genes with logFC > 2.5), while 400 were under-expressed (logFC < 2.5), suggesting a higher systemic response of patients to the vaccine than to the infection. In addition, among those DEGs with the lowest significant values (<10^−3^) and logFC in the range >|2.5|, all genes but three were found to be under-expressed (logFC values ranging from −2.6 to 4.4), and from these three over-expressed genes, the glutathione S-transferase mu 1 (*GSTM1*) gene has by far the highest logFC value (*P*-adjusted value of 4.07 × 10^−5^ and logFC = 9.8).

When comparing RVinf in acute phase against RVinf in convalescence phase we detected 675 DEGs, all of them over-expressed in acute against convalescence samples. DEGs with the higher logFC (>2.5) correspond to genes that are involved in leukocyte mediated immunity process (GO:0002443; *P*-adjusted value = 7.06 × 10^−3^).

### Pathway Analysis

**Figure 3:**
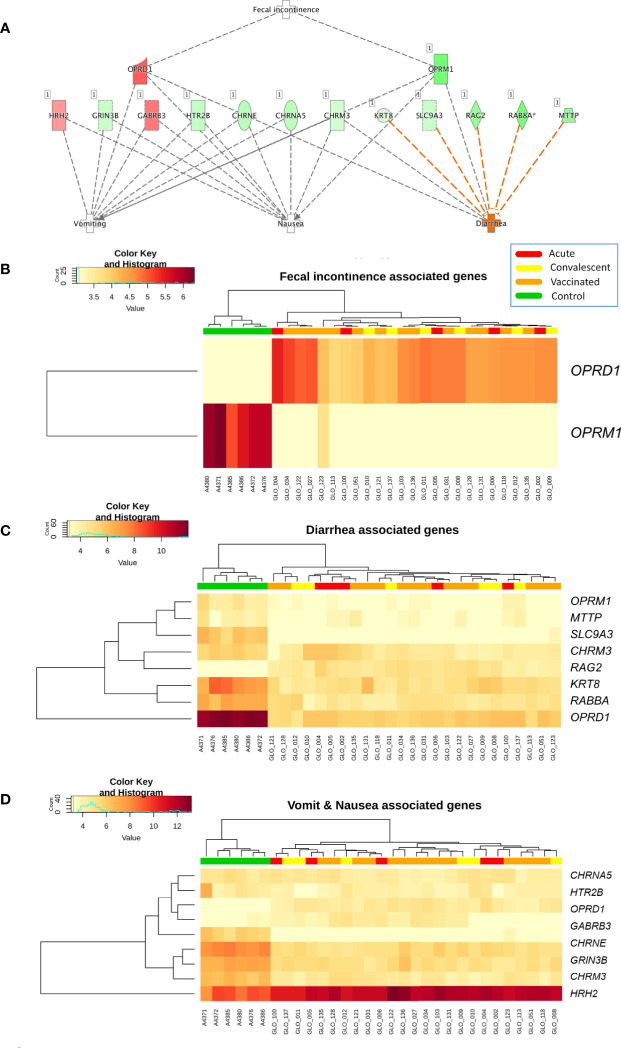
**(A)** Network of biomarkers associated to fecal incontinence, diarrhea, nausea, and vomiting within the genes differentially expressed between vaccinated and healthy controls according to IPA. The genes in red background are upregulated and the green ones are downregulated; **(B)** Two-way hierarchical clustering analysis heat map of the genes associated to fecal incontinence within the DEGs between vaccinated and healthy controls according to IPA; **(C)** Two-way hierarchical clustering analysis heat map of the genes associated to diarrhea within the DEGs between vaccinated and healthy controls according to IPA; and **(D)** Two-way hierarchical clustering analysis heat map of the genes associated to nausea and vomit within the genes differentially expressed between vaccinated and healthy controls according to IPA. See **Figure 2** for more information on color legend.

**Figure 4:**
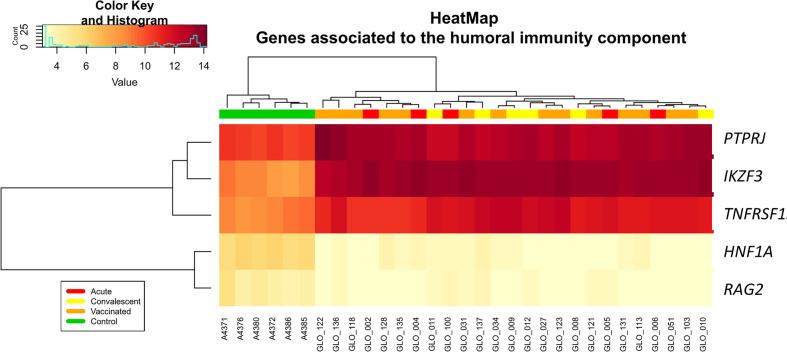
Two-way hierarchical clustering analysis heat map of genes associated with humoral immunity according to IPA. See **Figure 2** for more information on color legend.

Analysis of differential regulation using Ingenuity Pathway Analysis^®^ (IPA) showed that many of the DEGs in vaccinated *vs.* healthy children were associated with gastrointestinal disease, inflammatory disease, organ injury and abnormalities (*P*-value = 3.3 × 10^−4^; **Table S3** **;** **Figure 3**), including fecal incontinence (*P*-value = 3.1 × 10^−3^; **Figure 3B**), diarrhea (*P*-value = 1.7 × 10^−2^; **Figure 3C**), and nausea and vomiting (*P*-value = 2.7 × 10^−2^; **Figure 3D**). Two-way hierarchical clustering analysis heat maps highlights the differential expression patterns of the genes involves in these pathways between cohorts. Furthermore, IPA also identified a statistically significant over-expression of pathways and genes associated to the humoral immunity component of the adaptive immune system which is responsible for secreting antibodies with respect to controls (**Figure 4**) (*PTPRJ*, *IKZF3*, *TNFRSF1* genes). This result is consistent with the Fisher analysis showing that there is an enrichment in genes associated with the immune system in both comparisons RV5V *vs*. controls (*P*-value [Fisher exact test] = 5.5 × 10^−14^; OR = 2.12) and RVinf *vs*. controls (*P*-value [Fisher exact test] = 8.1 × 10^−15^; OR = 2.20).

In addition, IPA also identified (**Figure S1**) the pathway “abnormal morphology of midgut” (nine genes involved) as significantly enriched in RV5V *vs.* controls (*P*-value = 2.0 × 10^−3^); the heatmap of **Figure S1** shows the differential expression of these genes.

Enrichment of humoral immunity component of the adaptive immune system is also present when comparing RVinf against controls (**Figure 4**).

Overall, the results suggest that the activation of the immune system produced by the vaccine is comparable to the one caused by the wild type infection.

GO analysis indicates the over-expression of biomarkers associated with gastrointestinal injury and abnormalities (**Table S4**), including bacterial invasion of the epithelium (hsa05100, hsa05120) and a noticeable down-expression of genes associated to cell-to-cell adhesion: GO:0007155, GO:0022610, GO:0016337, hsa04540, hsa04530, hsa04520.

Furthermore, the pathway analysis results yielded by KEGG (**Table S5**) and GO ontology (**Table S4**) showed an enrichment of genes related to the regulation of cell cycle (hsa04110, GO:0051726, GO:0007049).

### Cell Deconvolution

Cell deconvolution analysis indicates a statistically significant increase of B and T lymphocytes in vaccinated children compared to controls (CD4T: *P*-value = 8.4 × 10^−5^; CD8T: *P*-value = 2.0 × 10^−3^; B cells: *P*-value = 5.0 × 10^−3^; **Figure S2**), in agreement with the IPA results. (**Table S3**).

The results also indicate that the relative proportion of innate and adaptive immune cells of the infected against the vaccinated children is statistically significant in several cell types (**Figure S2**); in particular when comparing convalescent-infected children against vaccinated.

### MiRNA Enrichment Analysis

**Figure 5:**
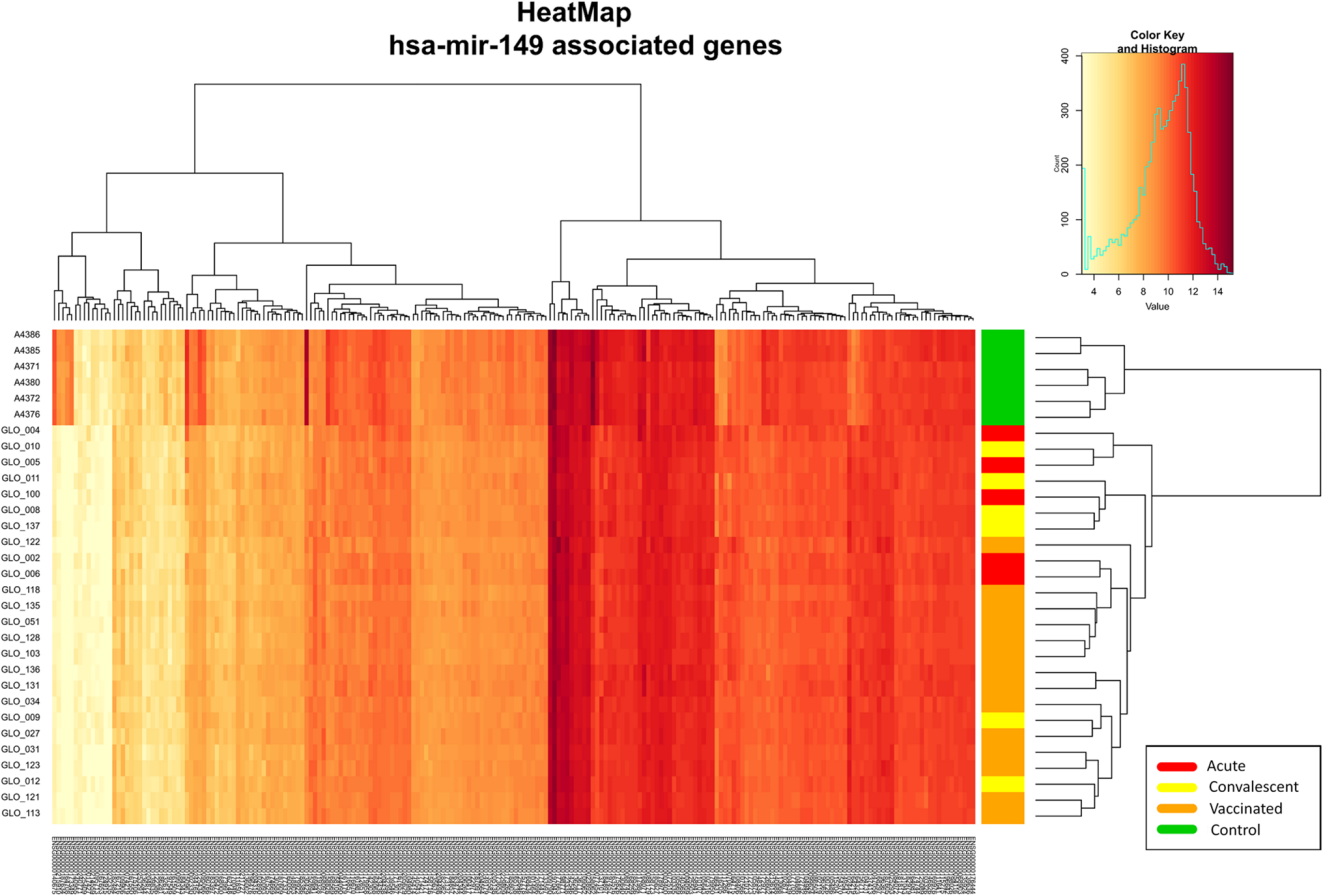
Two-way hierarchical clustering analysis heat map of the genes regulated by the miRNA hsa-mir-149 within the DEGs between vaccinated and healthy controls. See **Figure 2** for more information on color legend.

The association test for over-representation of microRNA-target between vaccinated children and controls yielded one remarkable result: from the 9,503 DEGs obtained, there were a total of 216 (**Figure 5**) that are targets of the microRNA hsa-mir-149 (*P*-value = 3.7 × 10^−2^; Expectation = 173; Observations = 216). It is worth mentioning that these target genes also showed differences between infected patients and controls in the two-way hierarchical clustering analysis heat maps.

### A Nine-Transcript RNA Signature to Differentiate Vaccinated *vs.* Unvaccinated

**Figure 6:**
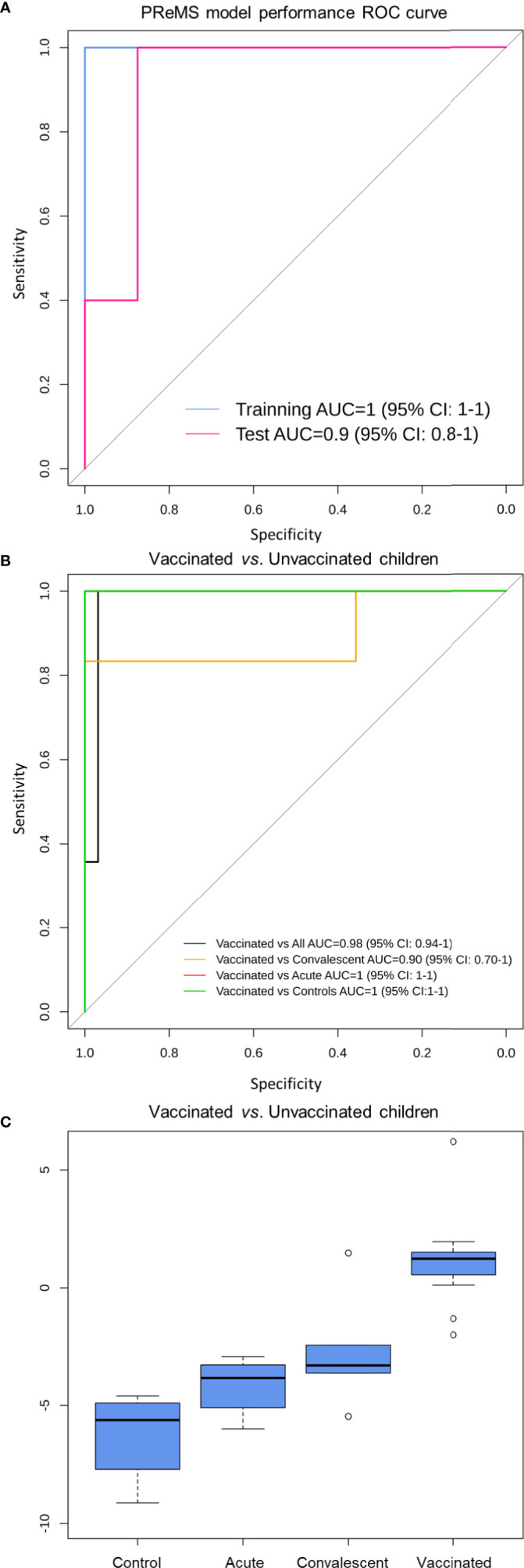
Classification performance to distinguish RV5 vaccinated group from control children based on a nine-transcript model. **(A)** ROC curve of the model to distinguish between vaccinated and unvaccinated children (including healthy controls, RV acute and convalescent children). The blue curve corresponds to the training set whereas the pink curve to the test set. **(B)** ROC curve of the model to distinguish between vaccinated, controls and convalescent children (note that some curves overlap). **(C)** Box and whisker plot of the model: the horizontal lines in the boxes indicate the median of each group; the lower and upper edges of boxes reflect interquartile ranges, and the whiskers are <1 times the interquartile range. Total score value from nine-transcript signature calculated as in (34–36) is represented in the y-axis.

We used the PReMS algorithm (31) to create the minimum gene signature able to distinguish between vaccinated and unvaccinated children (including healthy controls, RV acute and convalescent children). The algorithm found a nine-transcript signature (**Table 1**) that allows to accurately separate these three classes (**Figure 6A**). ROC curves indicate that our model differentiates correctly between vaccinated and unvaccinated group as the AUC was 100% for the training set and >90% for the test set (**Figure 6A**). When we look at the individual comparisons (**Figure 6B**) we found AUC values ranging from 100% (95%CI: 100–100) for vaccinated *vs*. RVinf in acute phase patients and vaccinated *vs*. controls, to 90% (95%CI: 70–100) for convalescent children *vs.* vaccinated groups. These latter two classes are more difficult to differentiate using this nine-transcript signature.

**Table 1:** Genes included in the nine-transcript signature.

Ensembl ID	Gene name	Gene	LR coefficient
ENSG00000118113	*MMP8*	Matrix metalloproteinase-8	-7.31×10^-03^
ENSG00000128512	*DOCK4*	Dedicator of cytokinesis 4	5.80×10^-03^
ENSG00000131142	*CCL25*	C-C motif chemokine ligand 25	-5.54×10^-02^
ENSG00000172738	*TMEM217*	Transmembrane protein 217	-9.37×10^-02^
ENSG00000175894	TSPEAR	Thrombospondin type laminin G domain and EAR repeat	2.41×10^-02^
ENSG00000196565	*HBG2*	Hemoglobin subunit gamma 2	-6.18×10^-06^
ENSG00000197768	*STPG3*	Sperm-tail PG-rich repeat containing 3	-2.41×10^-01^
ENSG00000198435	*NRARP*	Notch-regulated ankryrin repeat protein	-1.63×10^-01^
ENSG00000255423	*EBLN2*	Endogenous Bornavirus like nucleoprotein 2	2.24×10^-02^

Genes with positive logistic regression coefficient values are upregulated in vaccinated children relative to unvaccinated, whereas genes with negative values are downregulated. LR, logistic regression.

At the optimal cutpoint and according to the Youden statistic (−1.9967), the sensitivity was 100%; whereas the specificity was 97% with an AUC of 98% (95%CI: 94–100) when comparing vaccinated against unvaccinated children in the whole dataset.

Boxplot of the total score calculated from the nine-transcript signature in the different groups clearly shows its potential to differentiate between vaccinated cohort and those samples from infected and control subjects (**Figure 6C**).

## Discussion

RV vaccination causes global long-lasting changes in the transcriptome of peripheral blood cells, affecting the expression of more than 9,000 genes. Although the vast majority of children do not experience any adverse effects after vaccination (40), we found altered expression of biomarkers associated with vomit, diarrhea, fecal incontinence, and nausea. This suggests that the vaccine actually mimics a mild version of the disease.

Due to the reported association of intussusception and earlier RV vaccines in the past [risk of 1.5 [95%CI: 0.2–3.2] with the first dose according to Yih et al. (41)], large safety studies were conducted on the current vaccines RV5 and RV1 before they were approved. Nevertheless, the link between RV and intussusception remains unclear, up to the point that several studies have not found an increase in intussusception cases after administration of RotaTeq^®^ (42). There is now a general agreement in the medical community indicating that the benefits of RV vaccination substantially surpass the low risk of intussusception that might be associated with vaccination (43). We found that several DEGs between RV5V, and control children have been reported to be associated with intussusception (**Figure 2** **)** and abnormal morphology of midgut (**Figure S1**); *e.g.* gene *APC*, that is up-regulated in RV5V and RVinf, has been described to play a role in the development of a jejunal adenoma causing intussusception (29). This gene expression pattern may contribute to explain the reported increase of intussusception risk in vaccinated children. These genes could be targeted for the development of future safer vaccines and specifically analyzed in those children experiencing intussusception after vaccination. In addition, we have observed that *GSTM1* gene is among the DEGs with the most significant signal and was found to be strongly under-expressed in comparisons between RVinf acute children and vaccinated group. This gene encodes for a protein involved in detoxification of electrophilic compounds; the glutathione detoxifying system is important in maintaining intestinal barrier protection by attenuating enterocyte death (44). Glutathione S-transferase has been also previously proposed as a potential marker of intestinal epithelial cell damage (45).

Children vaccinated against RV over-expressed cell cycle related genes, this mechanism is used by many other viruses to facilitate their replication (46). Transcription of these genes may be a consequence of the increase of B2 lymphocytes observed in RV5V children (**Figure S2**; **Table S3**). As RV5 is a live-attenuated vaccine whose viral particles replicate in the gut, these results are in good agreement with our previous findings indicating that the host cell cycle is affected by RV infection (13, 47).

Previous studies suggested that antibody-based responses are necessary for acute control of RV infection, and for immunological memory (48). We found that vaccinated children have a significant increase in B cell proportion in peripheral blood (IPA analysis [**Table S3**]: *P*-value = 2.7 × 10^−2^; cell deconvolution analysis [**Figure S2**]: *P*-value = 5.0 × 10^−3^). This signal persists for a month after the last dose of the vaccine (the time the sample was taken in vaccinated children), in concordance with the role of B cells in long term protection against RV reinfections. Several studies claim that both B cells and CD8+-T cells play an important role in long term protection against RV reinfection (48–50). Consistently, our results also indicate that vaccinated children have higher levels of T cells (**Figures S2B, C**) compared to the healthy controls. Furthermore, vaccinated children express biomarkers associated with the differentiation of pre-T lymphocytes *CEBPA*, *MYH11*, *RAG2* and T cell receptor signaling (hsa04660) (**Tables S3** and **S5**). Also interesting is the fact that in general the innate and adaptive response of convalescent infected children seem to be more remarkable that the response provoked by the vaccine (see B-cells and natural killer in **Figure S2**); this can be due to (*i*) the stronger impact on the immune system of the wild infection compared to the vaccine, and/or (*ii*) the fact that the sampling time point for convalescent is about 3.7 months while for vaccinated children is roughly 5.2 months. In this time period, we cannot discard the possibility of new infections among convalescents. It is expected however, that such reinfections would modify the transcriptome in the same direction as the transcriptome of acute infected children; actually, this might be the case of one of the convalescent children in the PCA plot (see yellow dot within the cluster of infected children; **Figure 1B**).

Response to RV vaccination is also characterized by an over-expression of genes associated with gastrointestinal disease and inflammation (**Table S3**). RV5, like the RV, has a lytic cycle that burst epithelial cells to liberate the viral particles. Therefore, the presence of those biomarkers in vaccinated children possibly reflects that the intestinal barrier is being compromised due to the attenuated RV virus replication. This hypothesis is also supported by the fact that several pathways associated to cell–cell adhesion (*e.g.* GO:0007155, GO:0016337, hsa04530, hsa04520, hsa04540) are significantly down-regulated in the vaccinated cohort (**Tables S4** and **S5**).

Bioinformatic miRNA target enrichment analysis showed that the expression levels of >200 DEGs between RV5V and healthy controls (**Figure 5**) can be explained by the regulatory effects of the miRNA hsa-mir-149. Hsa-mir-149 is known to target the HIV gene *Vpr* (51) and also RV genes (52). Most recently, it has been described that hsa-mir-149 is able to significantly reduce polio replication within host cells (53). Further investigation of the relationship between RV and host mirna hsa-mir-149 may elucidate mechanisms of RV pathogenesis.

While RV5 is an oral vaccine containing reassorted RV strains that replicate poorly in the gut, we were able to see its effects in the blood transcriptome. This fact strengthens the hypothesis that RV causes a systematic infection, rather than one limited to the intestine (54, 55).

The PReMS method yielded a nine-transcript signature that distinguished vaccinated and unvaccinated children with an accuracy ~90%. Although the signature shows a good performance in the training and test sets, it would be necessary to validate this signature in an external cohort of vaccinated children. A signature that identifies children who have mounted a successful vaccine response might be of particular interest to detect vaccine failures, to prevent severe RV reinfections, to perform epidemiological control, and to evaluate immune response in the development of new RV vaccines. While the number of transcripts might be too large for a ready to use qPCR assay (36), other technologies would allow to easily test a 9-transcript panel that could be used for epidemiological surveillance or vaccine research purposes.

The present study has a few limitations: *i*) the results were derived from a limited number of subjects, even though the sample size lies within the standard range of transcriptome functional studies (56); *ii*) there is a lack of serological information of patients that might be useful for a more complete comprehension of the transcriptomic findings; *iii*) the results represent a cohort of South-European origin; therefore, additional analysis should ideally be carried out in other cohorts under the assumption that vaccines effectiveness could vary significantly *e.g.* in patients from low- and middle-income countries (57) or when considering other ancestral backgrounds (25); and *iv*) our results were obtained with children vaccinated with the RV5 vaccine (the only one available at the time of sample recruitment); therefore, it would be convenient to explore the impact of other RV vaccines on transcriptome. Finally, we analyzed the transcriptome of peripheral blood samples, away from the principal target of infection on the intestinal epithelium; therefore, it would be of particular interest to compare the impact of RV vaccination on these different tissues.

To conclude, the response to RV vaccination is characterized by the over-expression of genes associated with gastrointestinal disease, inflammation, activation of the immune system and gene over-expression of the cell cycle. Although the alterations of the transcriptome caused by RV vaccination strongly resemble the ones caused by community-acquired disease, there are DEGs that allow accurate discrimination of vaccinated and acute/convalescent infected children. Further research on these differences may help to unravel the molecular mechanisms of immune protection against RV, heterologous effects of the vaccine (58), and key features that allow the development of safer and more effective vaccines and novel antiviral drugs. Finally, we describe a nine-transcript signature/panel able to distinguish vaccinated children from unvaccinated, which may aid in the detection of vaccination failures.

## Data Availability Statement

The datasets presented in this study can be found in online repositories. The names of the repository/repositories and accession number(s) can be found below: European Nucleotide Archive, study accession number is: PRJEB41347.

## Ethics Statement

The studies involving human participants were reviewed and approved by the Ethical Committee of Clinical Investigation of Galicia (CEIC ref. 2012/301). Written informed consent to participate in this study was provided by the participants’ legal guardian/next of kin.

## Author Contributions

AS and FM-T conceived the study and gave financial support to the project. AG-C, MC-L, MC-T, SP, and J-GR were involved in sampling recruitment and laboratory analyses. AS, RB-A, DH-C, JH, and MK were involved in the data analysis. AS, RB-A, and AG-C wrote the first draft of the manuscript, which was revised by FM, MK, and JH. All authors contributed to the article and approved the submitted version.

## Funding

This study received support from projects: GePEM (Instituto de Salud Carlos III(ISCIII)/PI16/01478/Cofinanciado FEDER), DIAVIR (Instituto de Salud Carlos III(ISCIII)/DTS19/00049/Cofinanciado FEDER, Proyecto de Desarrollo Tecnológico en Salud), Resvi-Omics (Instituto de Salud Carlos III(ISCIII)/PI19/01039/Cofinanciado FEDER), BI-BACVIR (PRIS-3; Agencia de Conocimiento en Salud (ACIS)—Servicio Gallego de Salud (SERGAS)—Xunta de Galicia; Spain), Programa Traslaciona Covid-19 (ACIS—Servicio Gallego de Salud (SERGAS)—Xunta de Galicia; Spain) and Axencia Galega de Innovación (GAIN; IN607B 2020/08—Xunta de Galicia; Spain) awarded to AS, and projects ReSVinext (Instituto de Salud Carlos III(ISCIII)/PI16/01569/Cofinanciado FEDER), and Enterogen (Instituto de Salud Carlos III(ISCIII)/PI19/01090/Cofinanciado FEDER) awarded to FM-T.

## Conflict of Interest

FM-T has received honoraria from GSK, Pfizer, Sanofi Pasteur, MSD, Seqirus, and Janssen for taking part in advisory boards and expert meetings, and for acting as speaker in congresses outside the scope of the submitted work. JG-R has received honoraria from GSK, Pfizer, and MSD for taking part in advisory boards and expert meetings, and for acting as speaker in congresses outside the scope of the submitted work. FM-T has also acted as principal investigator in RCTs of the above-mentioned companies as well as Ablynx, Regeneron, Roche, Abbott, Novavax, and MedImmune, with honoraria paid to his institution.

The remaining authors declares that the research was conducted in the absence of any commercial or financial relationships that could be construed as a potential conflict of interest.

## Supplementary Material

Supplementary Figure 1 Two-way hierarchical clustering analysis heat map of genes associated to abnormal morphology of the midgut according to IPA. Each row represents one transcript; each column represents one patient, with a red bar above indicating the sample status red (acute), yellow (convalescent), orange (vaccinated), green (control). Expression intensity is indicated by color (high expression in red; low expression in yellow).

Supplementary Figure 2 Box and whiskers plots of the proportion of blood cells according to cell deconvolution analysis. **(A)** Dendritic cells, **(B)** CD4T lymphocytes, **(C)** CD8T lymphocytes, **(D)** plasma cells, **(E)** monocytes, **(F)** natural killer cells, **(G)** B lymphocytes, and **(H)** neutrophils. For clarity, statistically significant values are only given for comparisons between all conditions (acute and convalescent infected and healthy controls) against vaccinated children.

Supplementary Table 1 Detailed sample information.

Supplementary Table 2 Differentially expressed genes between controls and vaccinated/RV infected according to *Deseq2* corrected by age and gender.

Supplementary Table 3 Ingenuity canonical pathway analysis of the differentially expressed genes between controls and vaccinated children. The top diseases and functions are indicated as well as a detailed list of pathways specifically related to gastrointestinal and immunological diseases.

Supplementary Table 4 KEGG pathway enrichment analysis of the differentially expressed genes between controls and vaccinated children.

Supplementary Table 5 GO pathway enrichment analysis of the differentially expressed genes between controls and vaccinated children.
