# Supplementary figures and images for: Host Transcriptomic Response Following Administration of Rotavirus Vaccine in Infants’ Mimics Wild Type Infection
Source: Front Immunol. 2021 Jan 21;11:580219. doi: 10.3389/fimmu.2020.580219 (PMC7859632; doi:10.3389/fimmu.2020.580219)

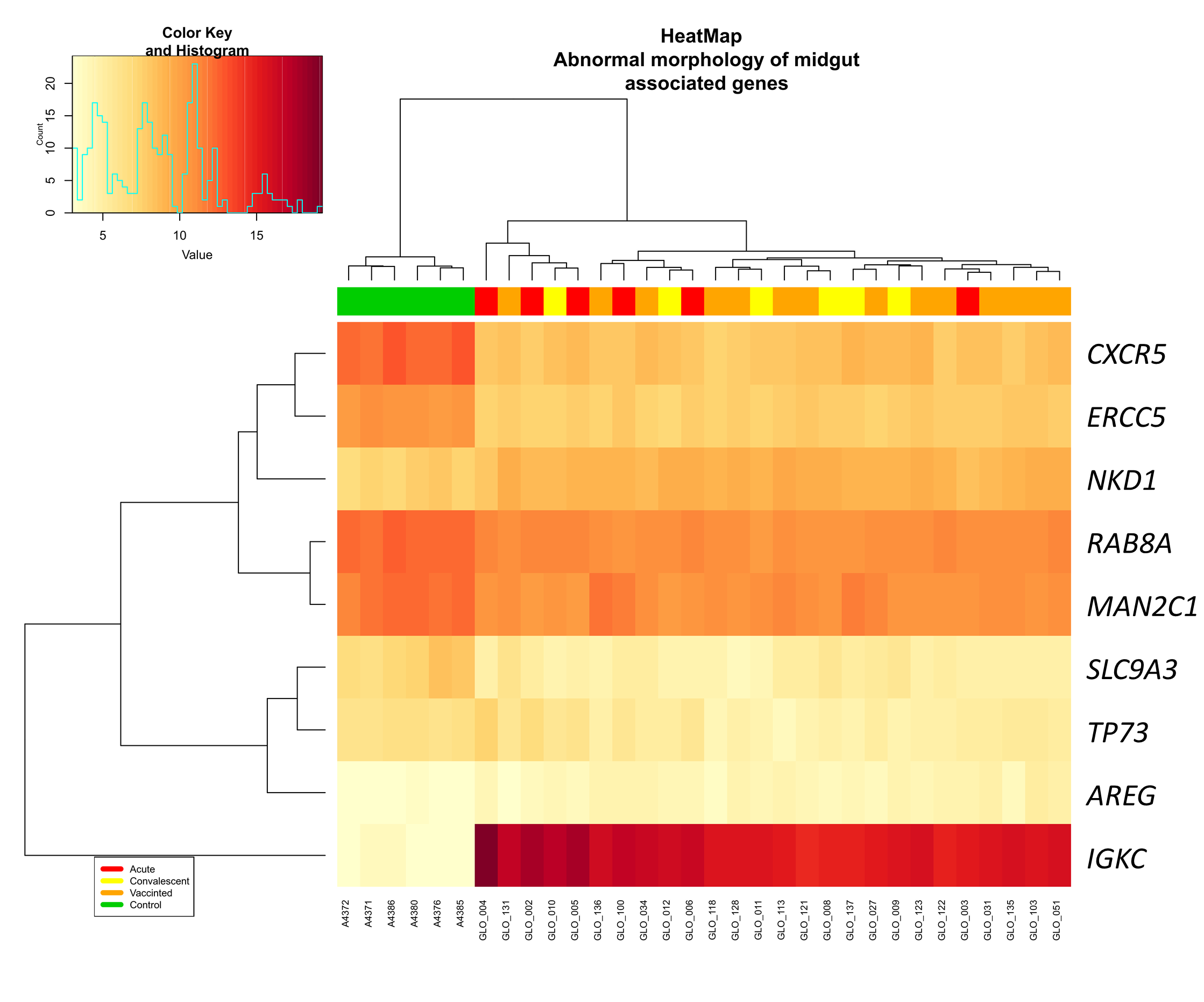

Supplement: Supplementary Figure 1 — Two-way hierarchical clustering analysis heat map of genes associated to abnormal morphology of the midgut according to IPA. Each row represents one transcript; each column represents one patient, with a red bar above indicating the sample status red (acute), yellow (convalescent), orange (vaccinated), green (control). Expression intensity is indicated by color (high expression in red; low expression in yellow). [file Image_1.tif]

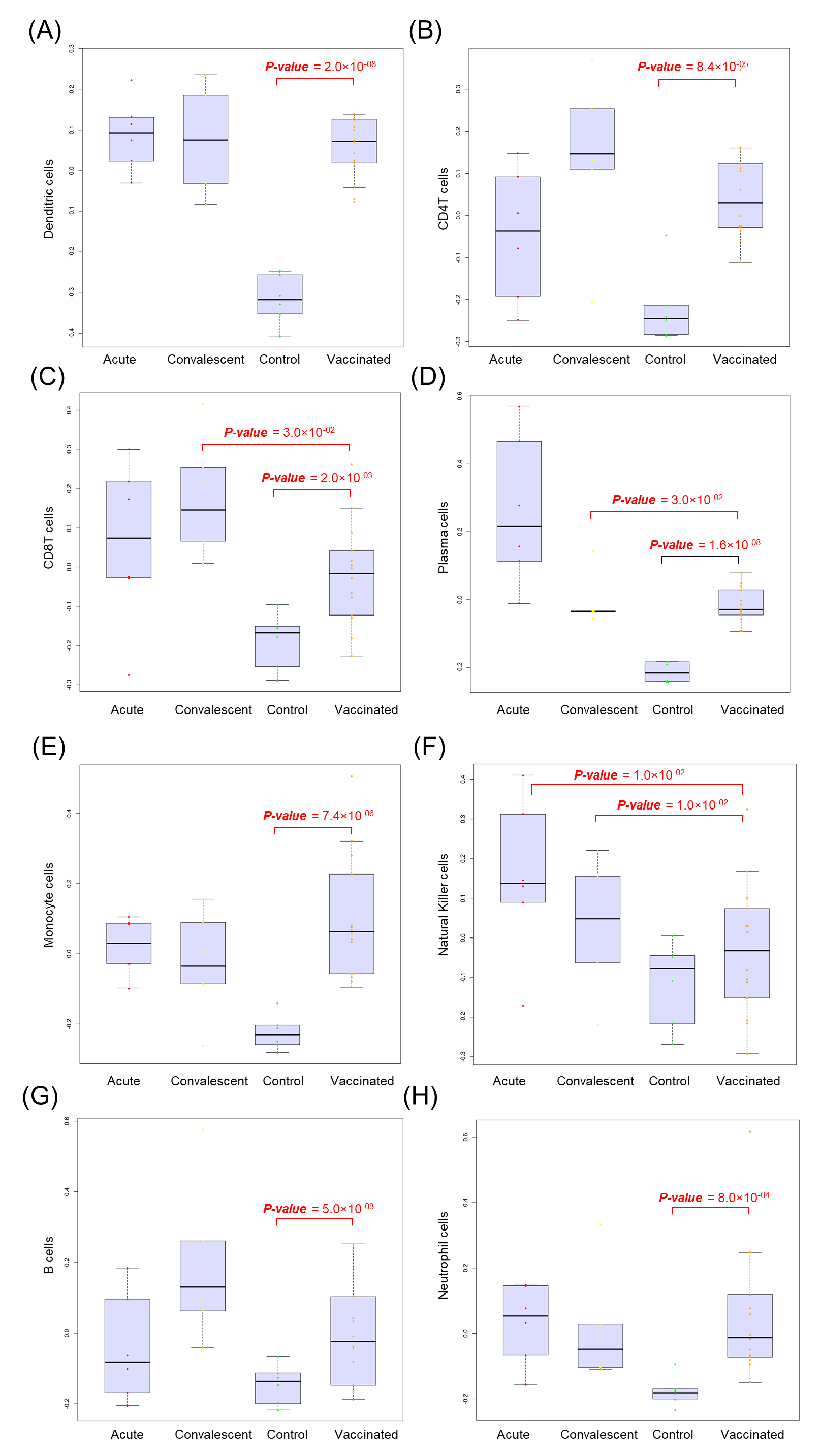

Supplement: Supplementary Figure 2 — Box and whiskers plots of the proportion of blood cells according to cell deconvolution analysis. (A) Dendritic cells, (B) CD4T lymphocytes, (C) CD8T lymphocytes, (D) plasma cells, (E) monocytes, (F) natural killer cells, (G) B lymphocytes, and (H) neutrophils. For clarity, statistically significant values are only given for comparisons between all conditions (acute and convalescent infected and healthy controls) against vaccinated children. [file Image_2.tif]
